# Effect of Calcium Salts on the Firmness and Physicochemical and Sensorial Properties of Iranian Black Olive Cultivars

**DOI:** 10.3390/foods12152970

**Published:** 2023-08-07

**Authors:** Mahnaz Ataollahi Eshkour, Azade Ghorbani-HasanSaraei, Ali Rafe, Seyed-Ahmad Shahidi, Shahram Naghizadeh Raeisi

**Affiliations:** 1 Department of Food Science and Technology, Ayatollah Amoli Branch, Islamic Azad University, Amol P.O. Box 66169-35391, Iran; 2 Department of Food Processing, Research Institute of Food Science and Technology (RIFST), Mashhad P.O. Box 91775-1163, Iran

**Keywords:** black olive, calcium salts, texture, phenolic compounds, Iranian cultivars

## Abstract

Black olive has become one of the most prestigious olives processed in the olive industry, and its processing has been increased recently in different countries. The firmness of black olives may be changed by the processing methods, fermentation, and solution salts. In this study, the employment of CaCl_2_, Ca-acetate, and Ca-lactate during the processing of some Iranian black olive cultivars, including Mari, Zard, Rowghani, Shengeh, Dakal, Dezful, and Fishomi, was evaluated in terms of physicochemical and phenolic compounds and textural attributes. The results showed that Ca-lactate improved the firmness of the Mari cultivar from 1455 to 1765 N/100 g in the pitted olive, and the same trend was obtained for the other cultivars. Ca-acetate improved the black shiny color of the Mari cultivar from 4.36 to 4.85 and the sensorial properties of the black olives, including gustatory and kinesthetic sensations, were improved by using a Ca-lactate solution. The application of calcium salts in the salt-free preservation solutions imparted neither bitterness to the olives nor discoloration. The highest amounts of acid (1.42–1.56%), fructose to mannitol ratio (1–1.2), and phenolic compounds (955–963 mg/kg) were found for the Zard cultivar. Furthermore, the residual content of oleuropein was higher when CaCl_2_ was employed (357 mg/kg). All of the calcium salts improved the firmness of the black olives, although the maximum firmness was observed for the Ca-lactate. Consequently, the formation of a black shiny color is related to the diffusion of phenolic compounds; however, this needs further investigation to determine which kind of phenolic compound is responsible for its black color.

## 1. Introduction

Olive fruit (*Olea Europaea*) is a species of small tree in the *Oleaceae* family traditionally found in the Mediterranean Basin. The annual production of the table olive is about 2.66 million metric tons according to the last report of the International Olive Council [1]. Although Spain is the main producer and exporter of the table olive (~35–40% of table olive production) [2,3], there are many other places where olives are cultivated and processed. Iran, by producing ~10,000 tons of virgin olive oil, is the easternmost producer country in the northern hemisphere. It imported almost 3500 tons and consumed 12,000 tons last year [1]. Olive cultivars are distributed across the provinces of Guilan, Zanjan, and Golestan in the north and Khozestan and Fars in the south of Iran. Moreover, most of the olives are utilized as green Spanish-style (approx. 60%), while black California-style olives are steadily increasing due to their attractive shiny color and healthy effects, which make them one of the most popular olive products for their application in pizzas, salads, and many other ready to eat products. However, while Iran has achieved a sustainable amount of commercialized olive products, due to wastewater environmental problems [4,5,6] and the use of olive oil [1], it has become stable in its production at 80,000 tons.

California-style olives are processed by exposing the green olives to an acidic solution followed by a series of lye (1–4% *w*/*v* NaOH or KOH) and oxygenated water baths over multiple days to remove bitterness [7]. As a result, the high amount of phenolic compounds, particularly the O-diphenol oleuropein, is decreased [6]. The most critical changes occur at the oxidation stage, which includes olive treatments with one to several lye solutions that penetrate progressively to the flesh, followed by washing to diminish the excess of alkali [8]. Then, the dark color is maintained and fixed by immersing the olives in a ferrous gluconate or lactate (0.1% *w*/*v*). Finally, the black olives are packed with a brine solution containing 2–4% NaCl and 10–40 mg/L ferrous salt to inhibit deterioration of the color [6,7,9].

Texture is one of the most important attributes in fermented products, such as cucumbers, peppers, capers, olives, and many other vegetables, in which the high amount of salt is used to maintain the firmness of the product and prevent the undesirable growth of microorganisms. Firmness can be considered as a measure of the supreme force at any time during the first compression cycle, and previous reports have shown that calcium organic salts can improve the firmness of the product [10,11]. For instance, the hardness of binary gels of an alginate/gum mixture has been improved when CaCl_2_ is used in pimento strip production [10].

Brine solutions, particularly high amounts of sodium chloride, have undesirable effects on health; therefore, several studies have been carried out to reduce salt and wastewater and to achieve improvements in nutritional effects [12,13,14,15,16,17]. American olive producers have authorized a salt-free preservation method for green olives for processing into black olives [18]. Due to the necessity of washing steps during the darkening of olives, an alternative procedure at aerobic conditions in an acidic solution has been also proposed [7,18]. However, the excess calcium addition is required to keep the firmness of the product; therefore, it is pivotal to evaluate the application of calcium salts (acetate and lactate) on different cultivars of olives. However, conflicting evidence on the usefulness of various calcium salts in delaying the softening of fruits and vegetables has been recorded; firmness was higher in lychee fruit, fresh-cut carrot, and mango treated with CaCl_2_ compared to treatment with calcium lactate [19,20,21,22,23], but fresh-cut melon showed the reverse effect [24], and canned peaches and fresh-cut cantaloupe showed no changes [22,24]. One of the primary disadvantages of calcium salts, particularly CaCl_2_, is that excessive quantities can give bitterness to food [22]. Bitterness was observed to be increased in black olives fermented with more than 2.6% CaCl_2_ [12], although this negative impression has not been seen in olives treated with a combination of calcium acetate and lactate evaluated at 32.3 mM each [25].

It has been understood that olive cultivars, the growing area, and the storage period influence the sensory profiles of black olives [6,26]. Furthermore, the flavor characterization of black olives as well as the textural and sensorial properties largely depend on the country of origin and the growing conditions [27]. No study has been performed on the black olive processing of Iranian olive cultivars to evaluate the firmness, phenolic compounds, or sensorial properties as functions of calcium salt solutions. Consequently, the aim of the current work was to evaluate the effect of CaCl_2_, calcium acetate, and lactate in California-style processing on the main cultivars of olives in Iran. Furthermore, firmness and sensory properties as the most important factors were also investigated. To the best of our knowledge, this is the first report on Iranian olive cultivars that can be applicable to comparisons with other olives, such as Manzanilla and Hojiblanca cultivars, in the world trade market.

## 2. Materials and Methods

### 2.1. Olive Processing

**Figure 1:**
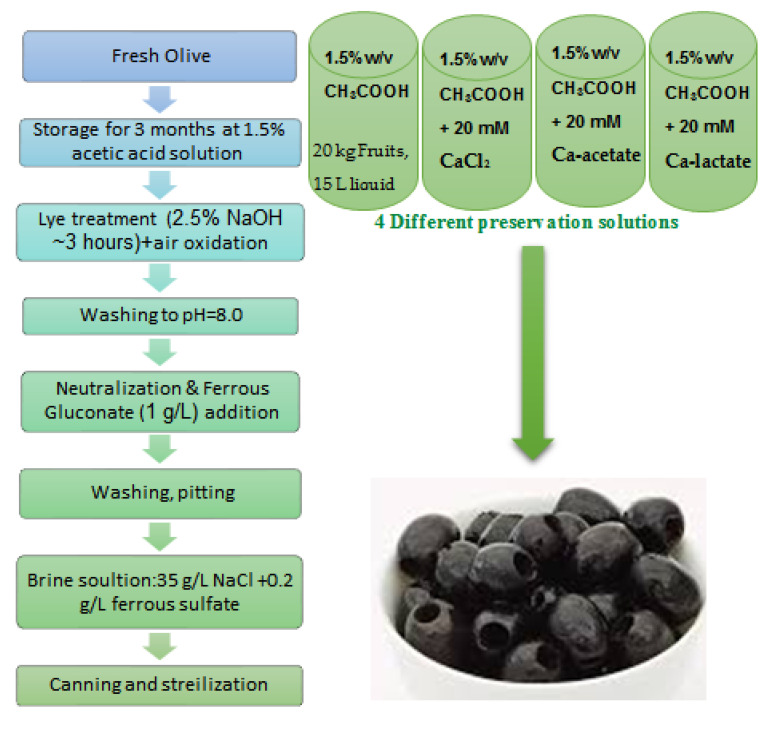
Processing flowchart of California-style black olive.

According to previous work on the Iranian olive cultivars, 7 traditional cultivars, including Mari, Zard, Rowghani, Shengeh, Fishomi, Dakal, and Dezful, were purchased from the market of three main olive cultivation regions (the Guilan, Golestan, and Fars provinces) at the end of October During the 2019/2020 olive season. The cultivars Mari, Zard, Rowghani, and Shengeh were obtained from Guilan province (north of Iran, 37°26′ N 49°33′ E), Shengeh and Fishomi were harvested from Golestan province (north of Iran, 37°20′ N 55°09′ E), and Dakal and Dezful were obtained from Fars province (south of the country, 29°25′ N 53°14′ E). An approximate amount of 10 kg of each cultivar was collected at the end of October during 2019/2020, and the samples after processing were divided into 0.5 kg in order to test for further examinations. Therefore, 20 batches of black olives from each cultivar were obtained and used for experimental analysis in at least three replicates. The processing of olives was performed according to García-Serrano’s procedure, with slight modifications (Figure 1) [28]. Briefly, fruits were harvested at their green color ripeness stage and stored in tanks for 3 months under acidic solution according to the method of de Castro [7]. Four different preservation solutions were used for varying cultivars: (i) blank; (ii) 20 mM CaCl_2_; (iii) 20 mM Ca-lactate; and (iv) 20 mM Ca-acetate in which 1.5% *w*/*v* acetic acid was used for all of the treatments. CaCl_2_, Ca-lactate, and Ca-acetate were supplied from Sigma (St. Louis, MO, USA). Then, they were transferred to the darkening and oxidation process with an alkaline solution. The lye was removed by washing until the pH of the water approached 8.0. Air was introduced into the tank’s bottom during the alkaline treatment and cleaning. A ferrous sulfate solution was applied to fix the color and to prevent it from fading. Finally, the olives were pitted and treated with a brine solution before being brought to the workplace and kept cold (Figure 1). After one month of storage, sensory and instrumental assessments were performed.

### 2.2. Physicochemical Analysis

The pH value, titratable acidity, and sodium salt were determined by a routine procedure [18,27]. Titratable acidity was measured by titrating up to pH 8.3 with 0.2 M NaOH and expressed as acetic acid. Titratable acidity was determined with 2 M HCl until the pH approached 2.6, and it was stated as the equivalent of NaOH per liter. NaCl was measured through back titration with silver nitrate. The analysis of organic acids and sugars was carried out using HPLC, which was the same as was used for phenolic compounds and is described hereinafter [7]. For sugar analysis, 0.5 mL of each sample was mixed with 1 g of the acidic resin Amberlite IR-120 and basic resin Amerlite IRS-93. An internal standard (1.5 mL of 0.5% **w*/*v** sorbitol) was also inserted, and the samples were occasionally shaken during 30 min. Then, 1 mL of the solution was centrifuged at 10,000× *g* for 5 min, and 20 µL was injected into the chromatograph. Organic acids were analyzed using a Spherisorb ODS-2 column (25 cm × 4.6 mm i.d., 5 µm) with distilled water as a mobile phase and a flow rate of 1.2 mL/min. An aliquot of 0.5 mL was diluted with distilled water (1:1) and centrifuged at 10,000× *g* for 5 min, and 20 µL was injected into the chromatograph.

According to Ramrez et al. (2015), a phenolic extract was generated by extracting the olive pulp with dimethyl sulfoxide (DMSO), followed by HPLC analysis [28]. Briefly, 0.1 g of olive fruits was mixed with 0.5 mL DMSO and sonicated for at least 5 min. The mixture was centrifuged at 5000× *g* for 5 min after resting for 30 min. For the assessment of phenolic chemicals, 0.25 mL of supernatant, 0.25 mL of syringic acid (2 mM), and 0.5 mL of deionized water were mixed together as an inner standard. It was put into the HPLC chromatograph after being filtered via a nylon filter with a pore size of 0.22 µm. HPLC was utilized to separate and quantify each chemical, with a Spherisorb ODS-2 column (25 cm × 4.6 mm i.d., 5 µm), an elution gradient of acidified water and methanol, a glide fee of 1 mL/min, and a temperature of 35 °C. The HPLC system consisted of a Waters 717 plus auto sampler, a column heater module, a 600 E pump, and a Waters 996 photodiode array detector operated with Empower software version 3.0 (Waters, Inc., Milford, MA, USA). Separation was obtained through elution using water (A) and methanol (B), for which the initial composition was 90% A and 10% B. After 10 min, the concentration of B was increased to 30% and kept for 20 min. Then, B was raised to 40% over 10 min, maintained for 5 min, and increased to 50%. Lastly, B was increased to 60, 70, and 100% in 5 min intervals. Chromatograms were recorded at 280 nm. All of these analyses were carried out on the fresh samples.

### 2.3. Calcium Measurements

In a boron–silicate tube, 1 g of olives and 1 mL of preservation fluid were combined with 25 mL of nitric acid and digested at 120 °C for eight hours. Then, 5 mL of an HClO_4_/HNO_3_ (4:1) solution was poured into the tube, and the nitric acid was extracted by heating at 140 °C. The leftover solution was placed in a graduated flask and filled with 25 mL of distilled water until the marker was reached. The sample was diluted with distilled water until the concentration neared 2–14 g/mL, and then lanthanum solution was added to achieve 5000 g/mL. A hollow (Ca, Mg, Cu, and Zn) multi-element cathode lamp was used in an atomic absorption spectrometer (GBC, model 932 Braeside, VIC, Australia).

### 2.4. Firmness

A Kramer shear-compression cell, which was coupled with a Texture Analyzer TA.TX plus (Stable Microsystems, Godalming, UK), was used to determine firmness according to previous work [10]. The mean of 10 replicate measurements was used as the firmness, each of which was performed on three pitted olives, and the firmness was expressed as Newton/100 g pitted olives. The machine measures the shear compression force in Newton (N) to break 3 pitted olives, and the crosshead speed was 200 mm/min.

### 2.5. Superficial Color

The surface color of the fruits was determined according to our previous work [29]. Briefly, surface photos of black olives were obtained with a digital camera (Canon EOS 1000D, Taiwan) with a resolution of (2272 × 1704 pixels) and recorded on a computer in JPEG format using software (Canon Utilities Zoom Browzer EX Version 6.1.1). Image J software version 1.40 g (National Institutes of Health, Bethesda, MD, USA) was used to increase the picture contrast using the Median filter. The RGB pictures were then transformed into L*a*b* units using Image J software version 1.40 g, and data in terms of L* (lightness, 0 (black) to 100 (white)), a* (+60 (red) to −60 (green)), and b* (+60 (yellow) to −60 (blue)) were produced. The following equation was used to compute the total color (E):

(1) $$∆E=\sqrt{{\left(L_{2}^{*}-L_{1}^{*}\right)}^{2}+{\left(a_{2}^{*}-a_{1}^{*}\right)}^{2}+{\left(b_{2}^{*}-b_{1}^{*}\right)}^{2}}$$

where subscribes 1 and 2 are for green and black olives, respectively.

Furthermore, the superficial color was also measured using a spectrophotometer (BYK-Gardner model 9000 Color-view, Silver Spring, MD, USA) with reflectance at 700 nm (R_700_). Any interference from stray light was minimized by covering the samples with a box that had a matte black interior. Lower reflectance values indicate darker fruit, and the results were the average of 10 determinations [18].

### 2.6. Sensorial Analysis

The olives were tested according to the “Method for sensory analysis of table olives”, COI/OT/MO 1/Rev.2 No 1 [30]. The olives were given to the trained panelists. Firstly, the panelists were trained to know the abnormal taste of the olive and the salty, bitter, acidic, hardness, fibrousness, and crunchiness sensations of the olive. Then, panelists were asked to make a grade based on the hedonic scale from 1 to 6. According to their results and through a comparison with our standard samples, the best panelists, who obtained the closest score to our standard score, were selected and used for the next step. A duo–trio test was used to determine the statistical difference among the samples. This approach commercially classifies olives by using characteristics linked to the perception of unpleasant feelings (‘abnormal taste’). Gustatory (salty, bitter, and acidic) and kinesthetic (hardness, fibrousness, and crunchiness) sensations were also evaluated. The attribute values were reported as the median of the individual data (9 trained testers), and the variability was given as the robust standard deviation. The obtained results were provided as mean± SD [31].

### 2.7. Statistical Analysis

Microsoft Excel 2010 (Microsoft Corporation, Redmond, WA, USA) and XLSTAT v. 2016 (Addinsoft, Paris, France) were used to assemble and compute all of the data. Statistical comparisons of mean values for each trial were performed using Sigmaplot software (version 8.0; Jandel Scientific, Corte Madera, CA, USA), followed by Duncan’s multiple range test (*p* < 0.05). Differences were judged significant in the sensory analysis when confidence intervals (*p* < 0.05) did not overlap.

## 3. Results and Discussion

### 3.1. Chemical Characteristics

The chemical characteristics of various cultivars of black olives with different salt treatments are given in Table 1. As can be observed, after 60 days, the pH varied between 3.6 and 4.13 units, falling short of the minimum of 4.3 units needed by the majority of producers to guarantee the shelf life of their olives. Most olives had an acidity of about 1.4% as % of acetic acid, although Rowghani fruit had a somewhat lower acidity (approximately 1.1%). The maximum pH (4.13) and acidity (1.63%) were observed for the Dezful variety, and the lowest values of pH (3.61) and acidity (1.38%) were reported for the Rowghani variety, which can be attributed to the higher amount of unsaturated fatty acids in Rowghani cultivars. These values are in line with previous studies of the Hojiblanca cultivar [17]. These findings are consistent with the concentration of lactic acid in these solutions, which was around 0.4% in tanks for the earlier cultivars but not yet found in tanks for the Hojiblanca cultivar [17]. In solutions of calcium acetate and calcium lactate, respectively, the Zard variety showed the highest amount of acetic and lactic acid. Furthermore, because the content of other sugars (glucose, fructose) was relatively low after 60 days, the highest fructose to mannitol ratio was noted for the Zard cultivars (1.2) of black olives (Table 1).

### 3.2. Phenolic Contents

Tyrosol, hydroxytyrosol, verbascoside, oleuropein, luteolin, and a few more minor chemicals were identified as the primary phenolic components in black olives. It was also discovered that the concentration of phenolic compounds in the preservation solutions of the Zard and Rowghani cultivars was not significantly higher than in those of other cultivars, with the exception of hydroxytyrosol and tyrosol (Table 2), but these compounds have not been reported as highly inhibitory against lactic acid bacteria growth [17]. It is also worth noting the residual oleuropein content in the Fishomi cultivar’s preservation solutions at 60 days, which must be connected to the higher concentration of this secoiridoid in the raw material of this cultivar compared to the Zard and Rowghani cultivars. When calcium chloride was used, the residual level of oleuropein was increased. Calcium slows the transfer of chemicals from olives to the surrounding liquids and vice versa [7]. As a result, slower diffusion of phenolic compounds was predicted in the presence of calcium during the preservation stage of black ripe olives. However, it had no influence on the course of olive fermentation, and no secondary rotting of the fruit was identified at any time.

### 3.3. Textural Attributes

The effect of different brine solutions as well as cultivars on the firmness of black olives after 2 months of storage is provided in Table 3. The Mari cultivar showed the highest firmness among the samples, and the firmness of the Shenghe (1746 N/100 g pitted olive) and Zard (1422 N/100 g pitted olive) cultivars was higher than the other cultivars. In comparison with the Spanish olives, the texture of the Mari and Zard cultivars was similar to the Manzanilla and Hojiblanca cultivars, respectively. The lower firmness of the Rowghani (1352) and Fishomi (1391) cultivars was obtained, which may be related to higher fat content and less pectin substances in their fruits. All of the calcium salts improved the firmness of the Iranian black olive cultivars, although there was a maximum firmness (1765) for the Ca-lactate treatments. Indeed, Ca-acetate and Ca-lactate solutions, due to the formation of acidic conditions in the brine solutions, protected the olives from softening during storage. The order of firmness in different brine solutions was: Ca-lactate > Ca-acetate > CaCl_2_ > control. Therefore, more softening was observed for the solution free of calcium ions. Similar findings have been found for the softening rate of olives under acidic conditions [17]. Accordingly, the addition of calcium into black olives is required to protect them from softening during storage, particularly when it is intended to satisfy the required strong texture of black olives to sustain their integrity for a longer time [7,32]. It should be noted that the maximum firmness of olives was observed for the Mari cultivar (1765), whereas the calcium levels were the same in all treated olives. Furthermore, black olives with calcium inclusion showed more firmness than those without calcium (control samples) during storage. On the other hand, the firmness effect of calcium salts on the black olives was independent from the cultivars. Although the effect of iron on the texture of olives was not investigated here, it is important to note that the presence of iron as ferrous sulfate slightly increased the firmness of the black olives, as examined in previous work [9].

In general, calcium ions contribute to the structural firmness, cell wall integrity, and cell turgor of fruits by generating cross-linkages between pectin molecules, thereby reinforcing and preventing plant cell collapse. The calcium content in the pulp of the olive at varying salt solutions confirmed its relationship with the firmness of the olive, although a particular trend for the calcium content in the different cultivars was not observed (Table 3). It was discovered that adding calcium to the preservation fluid allowed neutral sugars to be preserved in the cell wall of olive cultivars, resulting in reduced breakdown of the cell wall framework, and that this was connected to the firming action of calcium salts. On the other hand, as a result of the alkaline treatment, the pectins in black ripe olives are demethylated, resulting in a very sluggish softening rate [31]. As a result, the alkaline treatment is critical for the final product’s stiffness [5].

### 3.4. Superficial Color and Sensory Attributes

The shiny black color of the olive is a very critical quality property that mainly influences consumer acceptance. Different calcium salts did not exhibit discoloration or undesirable color in the olives (Table 3). Although there was a slight reduction in superficial color when the CaCl_2_ was used, the Ca-acetate and Ca-lactate improved the shiny black color of the olives. It has been shown that calcium ions postpone the diffusion of substances from olives to the surrounding liquid, and this could happen during the darkening stage [15]. Consequently, the formation of the black shiny color is related to the diffusion of phenolic compounds. Oxidations occur through the process, which may affect the color. However, considering the fact that the olives are black, the dominant pigments are anthocyanins, which may diffuse into the brine and may degrade, therefore showing a different trend than that of the usual phenols. However, although further investigation is required to determine which kinds of phenolic compounds are responsible for the black color, the Mari and Zard cultivars showed more color, and these have more tyrosol (122–166) than other cultivars.

The sensorial analysis exhibited that all black olives can be qualified as an “extra” category according to the low values of the negative sensations [31]. Furthermore, the results showed that none of the calcium salts at the level of 20 mM gave rise to bitterness in any of the Iranian olive cultivars (Table 4). However, it can be foreseen that this level of calcium concentration is lower than that of the cucumber pickle [33] and black olives [12]. Sensorial panelists have also understood the stronger hardness in olives preserved by calcium salts compared to the control, which is in line with objective experiments gained through texture analysis (Table 4). Furthermore, there is a slightly more kinesthetic sensation (hardness, fibrousness, and crunchiness) for the olives treated with Ca-acetate and Ca-lactate. Moreover, neither CaCl_2_, Ca-acetate, nor Ca-lactate influence the gustatory or kinesthetic characteristics of black olives after 2 months of storage. It should be emphasized once again that the concentration of calcium in the olive pulp has a significant impact on the diffusion of chemicals from the pulp to the liquid and vice versa. These findings regarding the sensory qualities of black olives are consistent with the sensory profiles of the Spanish cultivars Manzanilla and Hojiblanca [5,7].

## 4. Conclusions

It has been determined that the use of calcium salts, chloride, lactate, and acetate in the salt-free preservation solutions of black Iranian olives did not cause bitterness or discoloration. Furthermore, they can postpone softening and provide conditions for long-term preservation. The highest amounts of acid (1.63%), fructose to mannitol ratio (1.2), and phenolic compounds (hydroxytyrosol (973 mg/kg) and tyrosol (166 mg/kg)) were found for the Zard cultivars. Furthermore, because of the diffusion delay from the olives to the surrounding liquid and vice versa, the residual quantity of oleuropein (446 mg/kg) was greater when calcium chloride was used. In addition, more firmness was observed for the Mari (1765 N/100 g pitted olive) and Zard (1428 N/100 g pitted olive) cultivars than the other cultivars. All of the calcium salts improved the firmness of the Iranian black olive cultivars, although there was a maximum firmness for the Ca-lactate treatments. Indeed, Ca-acetate and Ca-lactate solutions, due to the formation of acidic conditions in the brine solutions, protected the olives from softening during storage. Calcium ions contribute to the structural hardness, cell wall integrity, and cell turgor of fruits by generating cross-linkages between pectin molecules, therefore reinforcing and preventing plant cell collapse. The calcium content in the pulp of the olive at varying salt solutions confirmed its relationship with the firmness of the olive, although a particular trend for the calcium content in the different cultivars was not observed. Consequently, the formation of a black shiny color is related to the diffusion of phenolic compounds. However, further investigation is needed to determine which kinds of phenolic compounds are responsible for the black color.

## Figures and Tables

**Table 1:** Chemical characteristics of the black olives of varying Iranian cvs. with different brine solutions after 2 months of storage *.

Salts	Parameters	Mari	Zard	Rowghani	Shengeh	Dakal	Dezful	Fishomi
Control	pH	3.97 ± 0.04 ^aA^	3.96 ± 0.05 ^aA^	3.61 ± 0.02 ^cB^	3.87 ± 0.06 ^bA^	3.86 ± 0.05 ^bA^	4.04 ± 0.03 ^aB^	3.85 ± 0.02 ^bA^
	Acidity (%)	1.52 ± 0.11 ^aA^	1.56 ± 0.23 ^aA^	1.57 ± 0.22 ^aA^	1.24 ± 0.10 ^dB^	1.38 ± 0.27 ^cB^	1.45 ± 0.05 ^bB^	1.27 ± 0.27 ^dC^
	Lactic acid (%)	0.36 ± 0.08 ^cB^	0.44 ± 0.11 ^bB^	0.54 ± 0.15 ^aB^	0.47 ± 0.19 ^bB^	0.38 ± 0.11 ^cB^	0.44 ± 0.08 ^bB^	0.34 ± 0.11 ^cC^
	Acetic acid (%)	1.30 ± 0.04 ^cB^	1.41 ± 0.12 ^bB^	1.53 ± 0.10 ^aB^	1.41 ± 0.15 ^bB^	1.36 ± 0.17 ^cB^	1.18 ± 0.09 ^dB^	1.09 ± 0.03 ^dB^
	Ethanol (%)	0.19 ± 0.06 ^bB^	0.24 ± 0.03 ^bA^	0.23 ± 0.05 ^bB^	0.28 ± 0.03 ^aA^	0.27 ± 0.07 ^aB^	0.16 ± 0.02 ^cA^	0.17 ± 0.03 ^dA^
	Fructose/mannitol ratio	0.51	1	0.6	0.42	0.75	0.08	0.05
CaCl_2_	pH	3.84 ± 0.02 ^bB^	3.82 ± 0.02 ^bB^	3.68 ± 0.03 ^eA^	3.77 ± 0.05 ^cB^	3.80 ± 0.02 ^cB^	4.06 ± 0.03 ^aB^	3.73 ± 0.02 ^dB^
	Acidity (%)	1.47 ± 0.32 ^bA^	1.45 ± 0.23 ^bB^	1.34 ± 0.17 ^dC^	1.38 ± 0.12 ^dA^	1.41 ± 0.17 ^cB^	1.57 ± 0.05 ^aA^	1.38 ± 0.19 ^dB^
	Lactic acid (%)	0.35 ± 0.07 ^bB^	0.37 ± 0.12 ^bB^	0.33 ± 0.15 ^cC^	0.36 ± 0.18 ^bC^	0.37 ± 0.07 ^bB^	0.38 ± 0.06 ^bB^	0.48 ± 0.06 ^aB^
	Acetic acid (%)	1.27 ± 0.06 ^cB^	1.42 ± 0.17 ^bB^	1.50 ± 0.11 ^aB^	1.41 ± 0.17 ^bB^	1.36 ± 0.17 ^cB^	1.18 ± 0.09 ^dB^	1.09 ± 0.03 ^dB^
	Ethanol (%)	0.21 ± 0.04 ^bB^	0.22 ± 0.04 ^bA^	0.28 ± 0.08 ^aA^	0.26 ± 0.04 ^aA^	0.26 ± 0.04 ^aB^	0.18 ± 0.01 ^cA^	0.13 ± 0.05 ^cA^
	Fructose/mannitol ratio	0.60	1.1	0.61	0.42	0.72	0.09	0.07
Ca-acetate	pH	3.78 ± 0.03 ^bC^	3.81 ± 0.05 ^bB^	3.62 ± 0.02 ^cB^	3.85 ± 0.03 ^bA^	3.78 ± 0.05 ^bB^	4.10 ± 0.03 ^aA^	3.76 ± 0.02 ^bB^
	Acidity (%)	1.46 ± 0.12 ^bA^	1.48 ± 0.13 ^bB^	1.46 ± 0.12 ^bB^	1.40 ± 0.16 ^cA^	1.49 ± 0.13 ^bA^	1.62 ± 0.04 ^aA^	1.44 ± 0.18 ^bA^
	Lactic acid (%)	0.34 ± 0.04 ^cB^	0.38 ± 0.11 ^cB^	0.44 ± 0.08 ^bB^	0.46 ± 0.22 ^aB^	0.38 ± 0.15 ^cB^	0.47 ± 0.05 ^aB^	0.42 ± 0.17 ^bB^
	Acetic acid (%)	1.67 ± 0.05 ^bA^	1.68 ± 0.11 ^bA^	1.78 ± 0.17 ^aA^	1.66 ± 0.08 ^bA^	1.45 ± 0.09 ^cA^	1.38 ± 0.11 ^cA^	1.28 ± 0.03 ^dA^
	Ethanol (%)	0.22 ± 0.03 ^bB^	0.22 ± 0.03 ^bA^	0.20 ± 0.07 ^cB^	0.27 ± 0.05 ^aA^	0.29 ± 0.05 ^aA^	0.17 ± 0.03 ^dA^	0.15 ± 0.02 ^dA^
	Fructose/mannitol ratio	0.52	1.2	0.62	0.44	0.74	0.10	0.08
Ca-lactate	pH	3.78 ± 0.02 ^cC^	3.79 ± 0.04 ^cB^	3.66 ± 0.02 ^dA^	3.88 ± 0.04 ^bA^	3.84 ± 0.03 ^bA^	4.13 ± 0.02 ^aA^	3.79 ± 0.02 ^cA^
	Acidity (%)	1.49 ± 0.11 ^bA^	1.52 ± 0.07 ^bA^	1.38 ± 0.16 ^dC^	1.37 ± 0.15 ^dA^	1.44 ± 0.15 ^cA^	1.63 ± 0.02 ^aA^	1.39 ± 0.12 ^dB^
	Lactic acid (%)	0.58 ± 0.11 ^cA^	0.68 ± 0.23 ^bA^	0.76 ± 0.08 ^aA^	0.66 ± 0.14 ^bA^	0.65 ± 0.10 ^bA^	0.67 ± 0.06 ^bA^	0.72 ± 0.13 ^aA^
	Acetic acid (%)	1.32 ± 0.04 ^cB^	1.41 ± 0.15 ^bB^	1.51 ± 0.14 ^aB^	1.38 ± 0.08 ^cC^	1.42 ± 0.17 ^bA^	1.27 ± 0.09 ^dC^	1.22 ± 0.03 ^dA^
	Ethanol (%)	0.27 ± 0.02 ^aA^	0.20 ± 0.04 ^bA^	0.21 ± 0.07 ^bB^	0.21 ± 0.07 ^bB^	0.23 ± 0.06 ^bB^	0.16 ± 0.02 ^cA^	0.18 ± 0.04 ^cA^
	Fructose/mannitol ratio	0.53	1.1	0.60	0.43	0.71	0.11	0.09

* Lowercase shows the statistical difference in different cultivars (rows), and uppercase shows the statistical difference among the varying salt solutions (columns) (*p* < 0.05).

**Table 2:** Phenolic compounds (mg/kg) of the black olives for varying Iranian cvs. with different brine solutions after 2 months of storage *.

Salts	Phenolic Compounds	Mari	Zard	Rowghani	Shengeh	Dakal	Dezful	Fishomi
Control	Hydroxytyrosol	971 ± 24 ^bA^	963 ± 15 ^bA^	987 ± 20 ^aA^	856 ± 33 ^dA^	847 ± 18 ^dA^	935 ± 14 ^cA^	967 ± 22 ^bA^
	Tyrosol	122 ± 11 ^bA^	116 ± 23 ^cA^	157 ± 22 ^aA^	124 ± 10 ^bA^	138 ± 27 ^bA^	145 ± 15 ^aA^	127 ± 23 ^bB^
	Verbascoside	42 ± 8 ^bB^	44 ± 11 ^bA^	54 ± 15 ^aA^	47 ± 19 ^aA^	42 ± 16 ^bA^	44 ± 18 ^bB^	43 ± 28 ^bB^
	Oleuropein	241 ± 9 ^dA^	333 ± 15 ^cA^	448 ± 11 ^bA^	361 ± 19 ^cA^	374 ± 27 ^cA^	250 ± 19 ^dA^	568 ± 43 ^aA^
	Luteolin	69 ± 16 ^cB^	62 ± 11 ^cA^	50 ± 25 ^cA^	60 ± 14 ^cA^	63 ± 18 ^cA^	76 ± 22 ^bA^	86 ± 18 ^aA^
CaCl_2_	Hydroxytyrosol	887 ± 16 ^dC^	958 ± 18 ^bA^	977 ± 34 ^aA^	868 ± 18 ^eA^	859 ± 24 ^eA^	937 ± 12 ^cA^	966 ± 21 ^aA^
	Tyrosol	113 ± 22 ^cB^	118 ± 16 ^cB^	149 ± 22 ^aA^	134 ± 10 ^bA^	128 ± 27 ^cA^	125 ± 18 ^cB^	133 ± 16 ^bA^
	Verbascoside	53 ± 12 ^aA^	35 ± 16 ^cB^	38 ± 11 ^cB^	56 ± 23 ^aA^	56 ± 12 ^aA^	49 ± 20 ^bA^	41 ± 16 ^bA^
	Oleuropein	239 ± 17 ^eA^	347 ± 18 ^cA^	446 ± 16 ^bA^	358 ± 24 ^cA^	363 ± 12 ^cA^	263 ± 32 ^cA^	574 ± 23 ^aA^
	Luteolin	71 ± 18 ^bA^	62 ± 16 ^cA^	51 ± 21 ^dA^	52 ± 18 ^dA^	61 ± 15 ^cA^	71 ± 17 ^bA^	89 ± 15 ^aA^
Ca-acetate	Hydroxytyrosol	897 ± 17 ^cC^	973 ± 16 ^aA^	970 ± 24 ^aA^	857 ± 21 ^dA^	864 ± 20 ^dA^	947 ± 18 ^bA^	976 ± 27 ^aA^
	Tyrosol	123 ± 17 ^bA^	117 ± 23 ^bB^	138 ± 22 ^aA^	128 ± 11 ^bA^	114 ± 19 ^bB^	121 ± 12 ^bB^	136 ± 28 ^aA^
	Verbascoside	55 ± 18 ^aA^	37 ± 10 ^bB^	42 ± 12 ^aA^	48 ± 18 ^aA^	55 ± 10 ^aA^	53 ± 17 ^aA^	54 ± 15 ^aA^
	Oleuropein	228 ± 14 ^eB^	340 ± 24 ^dA^	450 ± 14 ^bA^	360 ± 23 ^cA^	371 ± 20 ^cA^	248 ± 22 ^eB^	580 ± 16 ^aA^
	Luteolin	76 ± 11 ^aA^	61 ± 10 ^bA^	48 ± 22 ^cA^	56 ± 21 ^cA^	58 ± 12 ^bA^	68 ± 12 ^aA^	81 ± 11 ^aA^
Ca-lactate	Hydroxytyrosol	923 ± 16 ^cB^	955 ± 18 ^bA^	987 ± 12 ^aA^	863 ± 13 ^dA^	874 ± 19 ^dA^	938 ± 22 ^cA^	960 ± 12 ^bA^
	Tyrosol	106 ± 23 ^cB^	166 ± 30 ^aA^	127 ± 32 ^bB^	124 ± 27 ^bA^	108 ± 31 ^cC^	114 ± 16 ^cC^	133 ± 17 ^bA^
	Verbascoside	64 ± 19 ^aA^	57 ± 11 ^bA^	38 ± 24 ^cB^	53 ± 16 ^bA^	68 ± 12 ^aA^	51 ± 12 ^bA^	48 ± 14 ^cA^
	Oleuropein	258 ± 16 ^dA^	278 ± 13 ^dB^	452 ± 10 ^bA^	367 ± 17 ^cA^	368 ± 18 ^cA^	259 ± 29 ^dA^	568 ± 43 ^aA^
	Luteolin	82 ± 14 ^bA^	62 ± 13 ^bA^	56 ± 19 ^cA^	58 ± 20 ^cA^	65 ± 17 ^bA^	73 ± 21 ^aA^	76 ± 16 ^aB^

* Lowercase shows the statistical difference in different cultivars (rows), and uppercase shows the statistical difference among the varying salt solutions (columns) (*p* < 0.05).

**Table 3:** Color, firmness, and mineral salt contents of the black olives of varying Iranian cvs. with different brine solutions after 2 months of storage.

Salts	Properties *	Mari	Zard	Rowghani	Shengeh	Dakal	Dezful	Fishomi
Control	Firmness †	1455 ± 17 ^aD^	1394 ± 38 ^bC^	1364 ± 63 ^cB^	1422 ± 71 ^aD^	1379 ± 23 ^cD^	1265 ± 44 ^dD^	1391 ± 54 ^bC^
	Superficial color	4.67 ± 0.14 ^aB^	4.56 ± 0.09 ^bB^	3.78 ± 0.10 ^eC^	4.03 ± 0.17 ^dC^	4.43 ± 0.23 ^cC^	3.36 ± 0.11 ^eC^	3.27 ± 0.15 ^eB^
	∆E	20.74 ± 0.08 ^aB^	19.29 ± 0.21 ^bB^	19.42 ± 0.16 ^bC^	18.07 ± 0.27 ^cB^	17.59 ± 0.06 ^cB^	16.61 ± 0.16 ^dC^	17.43 ± 0.19 ^cB^
	Ca in flesh	612 ± 27 ^bD^	598 ± 36 ^cD^	593 ± 46 ^cC^	554 ± 29 ^cD^	481 ± 23 ^dC^	763 ± 42 ^aD^	645 ± 58 ^bC^
CaCl_2_	Firmness	1549 ± 22 ^aC^	1417 ± 27 ^bB^	1352 ± 16 ^cB^	1567 ± 22 ^aC^	1387 ± 18 ^cC^	1320 ± 25 ^dC^	1401 ± 38 ^bC^
	Superficial color	4.36 ± 0.17 ^aC^	4.31 ± 0.15 ^aC^	3.55 ± 0.23 ^cD^	3.85 ± 0.20 ^bD^	4.29 ± 0.09 ^aD^	3.23 ± 0.08 ^dD^	3.08 ± 0.09 ^eC^
	∆E	19.43 ± 0.15 ^aC^	18.55 ± 0.14 ^bC^	18.55 ± 0.27 ^bD^	17.85 ± 0.31 ^cC^	16.43 ± 0.15 ^dC^	15.44 ± 0.25 ^eD^	16.54 ± 0.08 ^dC^
	Ca in flesh	926 ± 15 ^aC^	876 ± 32 ^bC^	748 ± 27 ^dB^	773 ± 42 ^dC^	687 ± 35 ^eB^	825 ± 22 ^cC^	735 ± 18 ^dB^
Ca-acetate	Firmness	1687 ± 13 ^aB^	1407 ± 27 ^cB^	1363 ± 23 ^dA^	1588 ± 25 ^bB^	1406 ± 15 ^cB^	1378 ± 26 ^cA^	1416 ± 22 ^cB^
	Superficial color	4.85 ± 0.11 ^aA^	4.77 ± 0.13 ^bA^	4.16 ± 0.07 ^dA^	4.14 ± 0.06 ^dA^	4.53 ± 0.15 ^cB^	3.57 ± 0.15 ^eB^	3.50 ± 0.27 ^eA^
	∆E	24.55 ± 0.17 ^aA^	22.17 ± 0.16 ^bA^	20.96 ± 0.18 ^cB^	19.52 ± 0.11 ^cA^	18.49 ± 0.24 ^dA^	17.58 ± 0.22 ^eB^	18.48 ± 0.26 ^dA^
	Ca in flesh	967 ± 52 ^aB^	913 ± 18 ^bB^	745 ± 41 ^dB^	854 ± 29 ^cB^	796 ± 26 ^dA^	957 ± 34 ^aB^	823 ± 26 ^cA^
Ca-lactate	Firmness	1765 ± 23 ^aA^	1428 ± 18 ^bA^	1378 ± 42 ^cA^	1746 ± 37 ^aA^	1421 ± 11 ^bA^	1358 ± 14 ^cB^	1448 ± 28 ^bA^
	Superficial color	4.77 ± 0.22 ^aB^	4.63 ± 0.08 ^aA^	3.96 ± 0.12 ^bB^	4.11 ± 0.10 ^bA^	4.66 ± 0.22 ^aA^	3.63 ± 0.07 ^cA^	3.44 ± 0.12 ^dA^
	∆E	23.45 ± 0.16 ^aA^	21.08 ± 0.09 ^bA^	21.31 ± 0.12 ^bA^	19.36 ± 0.21 ^cA^	18.27 ± 0.16 ^dA^	17.87 ± 0.34 ^eA^	18.67 ± 0.21 ^dA^
	Ca in flesh	989 ± 43 ^aA^	968 ± 37 ^bA^	768 ± 29 ^dA^	827 ± 35 ^cA^	788 ± 19 ^dA^	968 ± 27 ^bA^	847 ± 36 ^cA^

* Lowercase shows the statistical difference in different cultivars (rows), and uppercase shows the statistical difference among the varying salt solutions (columns) (*p* < 0.05). †. Firmness was considered as N/100 g pitted olive, superficial color was measured at R700, and calcium was determined in ppm.

**Table 4:** Sensory analysis of black ripe olives of varying Iranian cvs. after 2 months of storage at room temperature *.

Cultivars	Brine Solutions	Negative Sensation	Gustatory Sensations			Kinesthetic Sensations		
		Abnormal Flavor	Salty	Bitter	Acid	Hardness	Fibrousness	Crunchiness
Mari	Control	1.2 ± 0.1	5.0 ± 0.2 ^a^	1.2 ± 0.1 ^b^	1.2 ± 0.2 ^b^	4.9 ± 0.3 ^c^	4.8 ± 0.2 ^b^	4.2 ± 0.1
	CaCl_2_	1.1 ± 0.2	5.8 ± 0.3 ^a^	1.8 ± 0.2 ^a^	1.3 ± 0.1 ^b^	5.7 ± 0.2 ^a^	5.2 ± 0.4 ^a^	4.7 ± 0.3
	Ca-acetate	0.9 ± 0.1	5.5 ± 0.2 ^a^	1.1 ± 0.2 ^b^	1.8 ± 0.2 ^a^	5.2 ± 0.2 ^b^	4.8 ± 0.1 ^b^	4.6 ± 0.1
	Ca-lactate	0.8 ± 0.1	5.4 ± 0.1 ^a^	1.2 ± 0.1 ^b^	1.6 ± 0.2 ^a^	5.3 ± 0.1 ^b^	4.6 ± 0.1 ^b^	4.5 ± 0.2
Zard	Control	1.1 ± 0.2	5.8 ± 0.3 ^a^	1.2 ± 0.1 ^b^	1.1 ± 0.2 ^b^	4.9 ± 0.3 ^c^	4.8 ± 0.2 ^b^	4.2 ± 0.1
	CaCl_2_	1.0 ± 0.1	5.2 ± 0.4 ^b^	1.8 ± 0.2 ^a^	1.2 ± 0.1 ^b^	4.7 ± 0.2 ^a^	5.2 ± 0.4 ^a^	4.7 ± 0.3
	Ca-acetate	0.9 ± 0.2	4.8 ± 0.1 ^b^	1.1 ± 0.2 ^b^	1.5 ± 0.2 ^a^	4.2 ± 0.2 ^b^	4.8 ± 0.1 ^b^	4.6 ± 0.1
	Ca-lactate	0.8 ± 0.2	4.6 ± 0.2 ^b^	1.2 ± 0.1 ^b^	1.3 ± 0.2 ^a^	4.3 ± 0.1 ^b^	4.6 ± 0.1 ^b^	4.5 ± 0.2
Rowghani	Control	1.1 ± 0.1	5.6 ± 0.2 ^a^	1.2 ± 0.1 ^b^	1.2 ± 0.2 ^b^	4.9 ± 0.3 ^c^	4.8 ± 0.2 ^b^	4.2 ± 0.1
	CaCl_2_	1.3 ± 0.2	4.7 ± 0.3 ^b^	1.8 ± 0.2 ^a^	1.1 ± 0.1 ^b^	4.7 ± 0.2 ^a^	5.2 ± 0.4 ^a^	4.7 ± 0.3
	Ca-acetate	1.4 ± 0.1	5.7 ± 0.1 ^a^	1.1 ± 0.2 ^b^	1.4 ± 0.2 ^a^	4.2 ± 0.2 ^b^	4.8 ± 0.1 ^b^	4.6 ± 0.1
	Ca-lactate	1.2 ± 0.3	5.2 ± 0.2 ^a^	1.2 ± 0.1 ^b^	1.3 ± 0.2 ^a^	4.3 ± 0.1 ^b^	4.6 ± 0.1 ^b^	4.5 ± 0.2
Shengeh	Control	1.1 ± 0.2	5.2 ± 0.4 ^a^	1.2 ± 0.1 ^b^	1.2 ± 0.2 ^b^	4.9 ± 0.3 ^c^	4.8 ± 0.2 ^b^	4.2 ± 0.1
	CaCl_2_	1.2 ± 0.1	5.1 ± 0.1 ^a^	1.8 ± 0.2 ^a^	1.1 ± 0.1 ^b^	4.7 ± 0.2 ^a^	5.2 ± 0.4 ^a^	4.7 ± 0.3
	Ca-acetate	1.0 ± 0.1	5.0 ± 0.2 ^a^	1.1 ± 0.2 ^b^	1.2 ± 0.2 ^b^	4.2 ± 0.2 ^b^	4.8 ± 0.1 ^b^	4.6 ± 0.1
	Ca-lactate	1.1 ± 0.1	4.8 ± 0.3 ^a^	1.2 ± 0.1 ^b^	1.4 ± 0.2 ^a^	4.3 ± 0.1 ^b^	4.6 ± 0.1 ^b^	4.5 ± 0.2
Dakal	Control	1.1 ± 0.1	5.1 ± 0.2 ^a^	1.2 ± 0.1 ^b^	1.2 ± 0.2 ^a^	4.9 ± 0.3 ^a^	4.8 ± 0.2 ^b^	4.2 ± 0.1
	CaCl_2_	1.1 ± 0.1	5.5 ± 0.2 ^a^	1.8 ± 0.2 ^a^	1.2 ± 0.1 ^a^	4.7 ± 0.2 ^b^	5.2 ± 0.4 ^a^	4.7 ± 0.3
	Ca-acetate	1.0 ± 0.2	5.4 ± 0.2 ^a^	1.1 ± 0.2 ^b^	1.1 ± 0.2 ^a^	4.2 ± 0.2 ^c^	4.8 ± 0.1 ^b^	4.6 ± 0.1
	Ca-lactate	0.9 ± 0.1	5.3 ± 0.4 ^a^	1.2 ± 0.1 ^b^	1.3 ± 0.2 ^a^	4.3 ± 0.1 ^c^	4.6 ± 0.1 ^b^	4.5 ± 0.2
Dezful	Control	1.0 ± 0.1	5.3 ± 0.2 ^a^	1.2 ± 0.1 ^b^	1.2 ± 0.2 ^b^	4.9 ± 0.3 ^a^	4.8 ± 0.2 ^b^	4.2 ± 0.1
	CaCl_2_	1.1 ± 0.1	5.2 ± 0.2 ^a^	1.8 ± 0.2 ^s^	1.3 ± 0.1 ^b^	4.7 ± 0.2 ^b^	5.2 ± 0.4 ^a^	4.7 ± 0.3
	Ca-acetate	0.9 ± 0.1	5.1 ± 0.2 ^a^	1.1 ± 0.2 ^b^	1.4 ± 0.2 ^a^	4.2 ± 0.2 ^c^	4.8 ± 0.1 ^b^	4.6 ± 0.1
	Ca-lactate	0.8 ± 0.1	5.1 ± 0.1 ^a^	1.2 ± 0.1 ^b^	1.2 ± 0.2 ^b^	4.3 ± 0.1 ^c^	4.6 ± 0.1 ^b^	4.5 ± 0.2
Fishomi	Control	1.1 ± 0.2	5.8 ± 0.3 ^a^	1.2 ± 0.1 ^b^	1.2 ± 0.2 ^a^	4.9 ± 0.3 ^a^	4.8 ± 0.2 ^b^	4.2 ± 0.1
	CaCl_2_	1.2 ± 0.1	5.6 ± 0.2 ^a^	1.8 ± 0.2 ^a^	1.1 ± 0.1 ^b^	4.7 ± 0.2 ^b^	5.2 ± 0.4 ^a^	4.7 ± 0.3
	Ca-acetate	1.2 ± 0.2	5.1 ± 0.2 ^b^	1.1 ± 0.2 ^b^	1.3 ± 0.2 ^a^	4.2 ± 0.2 ^c^	4.8 ± 0.1 ^b^	4.6 ± 0.1
	Ca-lactate	1.0 ± 0.1	5.2 ± 0.2 ^b^	1.2 ± 0.1 ^b^	1.2 ± 0.2 ^a^	4.3 ± 0.1 ^c^	4.6 ± 0.1 ^b^	4.5 ± 0.2

* For each cultivar in each column, median values followed by the same letter do not differ at the level of 5% significance because of superimposing their corresponding confidence. There is no statistical letter for abnormal flavor or crunchiness because all of the results are not statistically significantly different (*p* < 0.05).

## Data Availability

Data are contained within the article.
